# Genotypic Responses in Maize Germination Under Water and Thermal Stresses

**DOI:** 10.3390/plants15172624

**Published:** 2026-08-27

**Authors:** Rafael Cardoso Lourenço dos Anjos, Carla Gomes Machado, Edésio Fialho dos Reis, Dayton Henrique Alves Nogueira, Caíque Lopes Silva, Maria Eduarda Ferreira, Ingrid Maressa Hungria de Lima e Silva, Arthur Almeida Rodrigues, Juliana de Fátima Sales, Ricardo de Castro Dias, Nathália de Souza Paulino, Kamilla Morois Silveira, Simério Carlos Silva Cruz, Luciana Celeste Carneiro, Danielle Fabiola Pereira da Silva, Mayara Cristina Lopes, Givanildo Zildo da Silva

**Affiliations:** 1 Department Federal Institute of Education, Science and Technology (IF Goiano), Campus Rio Verde, P.O. Box 66, Rio Verde 75901-970, Goias, Brazil; arthuralmeidaeng@gmail.com (A.A.R.); juliana.sales@ifgoiano.edu.br (J.d.F.S.); 2 Institute of Agricultural Sciences, Federal University of Jataí, Jataí 75801-615, Goias, Brazil; carlagomesmachado@ufj.edu.br (C.G.M.); edesioreis@ufj.edu.br (E.F.d.R.); daytonhenrique@discente.ufj.edu.br (D.H.A.N.); caiquelsilva@discente.ufj.edu.br (C.L.S.); maeduarda.agro@gmail.com (M.E.F.); simerio@ufj.edu.br (S.C.S.C.); luciana_celeste_carneiro@ufj.edu.br (L.C.C.); daniellefpsilva@ufj.edu.br (D.F.P.d.S.); 3 Institute of Agricultural Sciences, Federal University of Lavras, Lavras 37200-900, Minas Gerais, Brazil; ingridm_hungria@hotmail.com; 4 Research and Extension Support Group (GAPE – ESALQ), Piracicaba 13418-900, São Paulo, Brazil; 5 Institute of Agricultural Sciences, University of Rio Verde, Rio Verde 75901-970, Goias, Brazil; nathalia.paulino@academico.unirv.edu.br (N.d.S.P.); kamilla.silveira@academico.unirv.edu.br (K.M.S.); mayara@unirv.edu.br (M.C.L.); givanildo@unirv.edu.br (G.Z.d.S.)

**Keywords:** climate change, varieties, open pollination, *Zea mays*, tolerant seeds, germination, initial seedling establishment

## Abstract

Water stress reduces germination and seedling emergence by compromising the metabolic activity of seeds. Associated with this, extreme thermal events have intensified, and temperatures above 30 °C can significantly reduce maize yield. In this scenario, the selection of tolerant genotypes is essential. This study evaluated the germination potential of 22 maize genotypes, including open-pollinated genotypes and commercial varieties. Seeds were characterized by moisture content, thousand-seed weight, germination, and emergence in the field. Subsequently, they were subjected to thermal stress of 5, 15, 25, 35, and 40 °C, through the adapted standard germination test. For water stress, the seeds were subjected to different water retention capacities (15, 30, 60, and 100%). The results indicated relevant genetic variation among the genotypes. For thermal stress, there was no germination at extreme temperatures (5 and 40 °C), while at 15 °C, there were significant differences between genotypes. At 35 °C, there was a reduction in vigor and an increase in abnormalities. Open-pollinated genotypes showed superior physiological performance under stress conditions. For water stress, at 15% there was low performance; at 30%, initial improvement; at 60%, ideal conditions with greater vigor; and at 100%, maintenance of performance, with little significant gains. Maize genotypes showed tolerance to water stress between 15% and 100% of the water retention capacity, with normal germination and seedling formation. Materials 3, 4, 8, 12, 16, and 20 showed greater tolerance to water restriction, while 2, 10, 14, 18, and 22 had better performance in more humid environments. Materials 7, 9, and 10 stood out for their greater vigor and germination. Regarding thermal stress, germination occurred between 15 °C and 35 °C, even outside the ideal range. Material 3 (AS1598) showed higher thermal tolerance, with better physiological performance, higher germination, and higher vigor.

## 1. Introduction

Maize (*Zea mays L*.) is one of the most important agricultural crops in the world, playing a strategic role in the production of food, feed, and biofuels. However, climatic variations, such as temperature and water availability, have negatively impacted maize yield. In this context, the importance of open-pollinated genotypes (OPG) of maize [1], widely used by small and medium-sized producers in human and animal diets [2], stands out.

Although maize is a C4 plant, efficient in the use of solar radiation and relatively tolerant to abiotic stresses, adverse conditions can compromise its development, reducing phenotypic plasticity and resulting in the formation of uneven ears [3]. However, the germination and seedling establishment phases receive less emphasis when compared to the final stages of the crop, for grain production or silage [4].

Water stress soon after sowing is one of the main factors that affect the germination process, interfering with the enzymatic activities of seeds and reducing seedling emergence. Thus, the development of genotypes tolerant to low water availability, through selection processes and physiological and genetic mechanisms, has become a promising alternative [5], especially considering that the genes responsible for tolerance to abiotic stresses are inherited from their parents [6].

Similarly, thermal stress, characterized by exposure to temperatures outside the ideal range (25 to 30 °C) established by the Rules for Seed Analysis—RAS [7], can compromise germination and initial seedling establishment in the field [3]. This scenario has intensified due to global warming, which has been promoting significant changes in agricultural systems. According to the Intergovernmental Panel on Climate Change [8], the increase in the global average temperature (1.5 and 2.0 °C), this being the average annual temperature increase, could compromise production by 2050. For the maize crop, temperatures above 30 °C are associated with yield reductions of 10–20%, an effect already observed in the Midwest region of Brazil [9].

In this climate context, global initiatives, such as COP30 (United Nations Conference on Climate Change), have driven actions aimed at mitigating the impacts of climate change on agricultural production, reinforcing the importance of studies aimed at evaluating the physiological quality and resistance of seeds to abiotic stresses [10,11].

In this scenario, it is essential to understand seed production systems. Open-pollinated genotypes have greater genetic variability and allow the reuse of seeds. On the other hand, commercial hybrids are obtained through controlled crosses between parental lines, requiring greater isolation and intensive management, such as detasseling, resulting in more uniform and productive seeds, but with higher production costs and the impossibility of reuse due to the loss of hybrid vigor and loss of resistance to stresses [12,13].

Despite scientific advances addressing climate-related issues, most studies available in the agricultural field focus on commercial hybrid seeds, resulting in research gaps involving genotypes still under development, such as open-pollinated genotypes (OPG), particularly regarding maize seed germination and early seedling establishment. Furthermore, the high genetic variability of these materials may facilitate the selection of genotypes that are better adapted and more tolerant to abiotic stresses, thereby contributing to plant breeding programs and the sustainability of agricultural production systems [14,15].

Considering that the use of high-quality and economically accessible seeds is essential for small producers, OPGs are a promising alternative, as they allow the selection of genotypes that are more tolerant to thermal variations and adapted to the edaphoclimatic conditions of the Midwest region, although they are still in the development phase [16,17].

Therefore, the objective of this study was to evaluate the germination potential of open-pollinated genotypes of maize, of the F_2_ generation, not commercialized and commercial materials, and under different conditions of temperature and water availability.

## 2. Results

### 2.1. Water Stress

In the characterization of the seeds, moisture content varied from 5% to 13%, while thousand-seed weight ranged between 236.87 g and 343.94 g. Germination ranged from 89% (open-pollinated genotype 19) to 98% (open-pollinated genotypes 3, 4, 6, 12, and 15).

No significant interaction was observed between genotypes and water retention capacity for the variables evaluated (Table 1). The variables germination, dead seeds, first emergence count, shoot length, and shoot dry mass were significantly affected by both factors separately. For abnormal seedlings, there was only an effect of water retention capacity, with a mean of 11% of abnormal seedlings. For germination, the genotypes were grouped into two distinct groups, with a mean of 84.1% (genotypes 12, 15, and 22) and a mean of 74.2% (genotypes 13, 14, and 16 to 21). For dead seeds, materials 1, 11, 13, 14, and 16 to 21 had a mean of 7.5%, while the others showed seed mortality of 2.5% (Table 1).

Three groups were formed for the first emergence count: materials 1 (71%) to 15 (69%) and 22 (72%); materials 17 (61%) to 20 (64%), and materials 16 (55%) and 12 (72%). Groups were also formed for the emergence speed index, for materials 6 (5.3), 7 (5.5), 9 (5.8) to 11 (5.5) and 22 (5.4), for materials 1 (4.9) to 5 (4.9), 8 (5.0), 12 (5.1) to 15.0, and for materials 16 (4.1) to 21 (4.1).

Shoot length showed a mean value of 17.8 cm for materials 1 to 4, 6, 7, 9 to 15, 17, 20, and 22, and a lower mean of 15.7 cm for materials 5, 8, 16, 18, 19, and 21. For shoot dry mass, there was a greater accumulation for materials 1 to 4, 6 to 15, and 17 to 19, with 0.044 g, followed by materials 5, 16, and 20, with a mean of 0.036 g, and finally, commercialized materials 21 and 22, with 0.036 g. For root dry mass (RDM), the group with the highest accumulation contained materials 1, 3, 5, 7 to 11, 13 to 15, and 18 (<0.230 g), while the others formed a secondary group with the lowest RDM accumulation.

**Figure 1:**
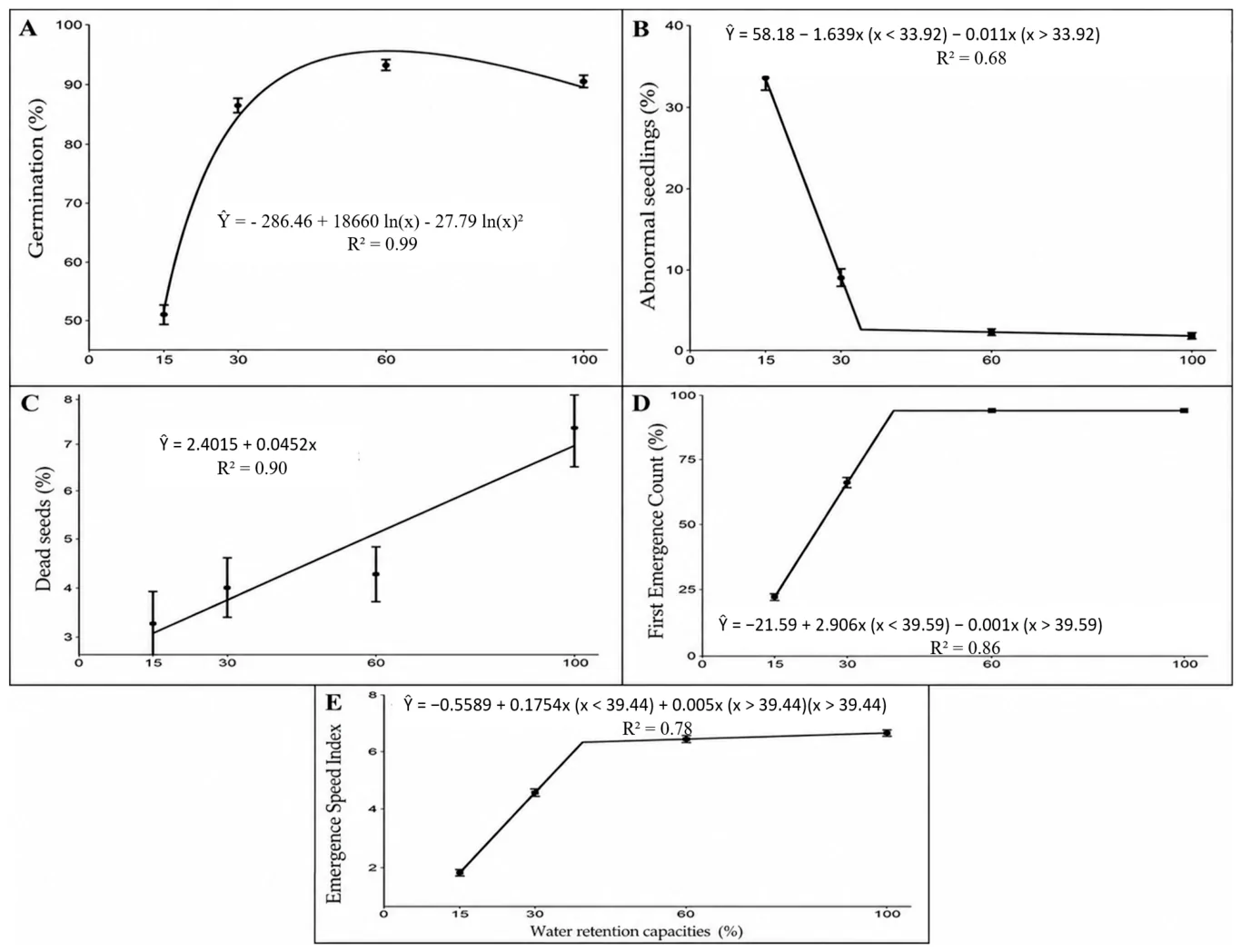
Mean values of germination (**A**); abnormal seedlings (**B**); dead seeds (**C**); first emergence count (**D**); and emergence speed index (**E**) of maize genotypes subjected to water retention capacities of 15, 30, 60, and 100%.

**Figure 2:**
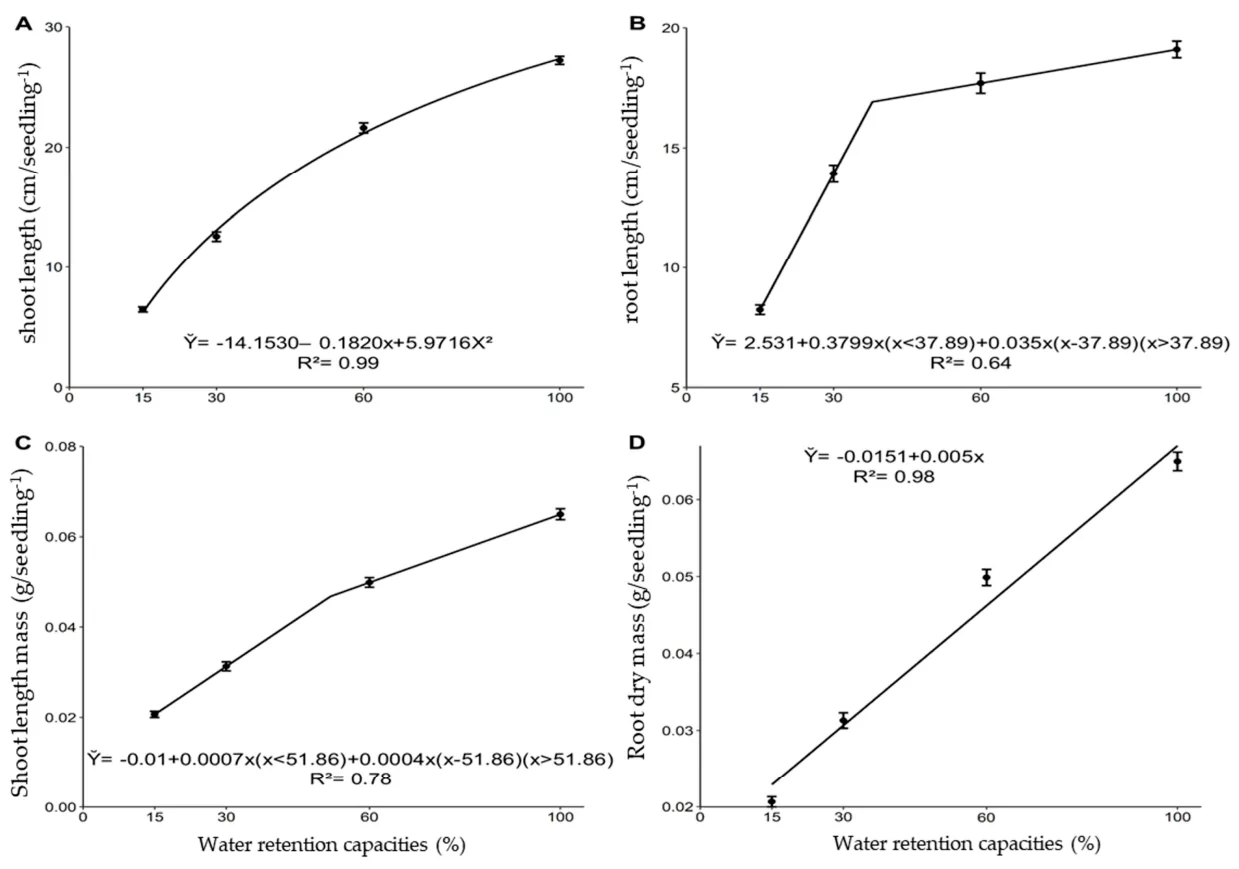
Values of shoot length (**A**), root length (**B**), shoot dry mass (**C**), and root dry mass (**D**) of seeds of maize materials subjected to water retention capacities of 15, 30, 60, and 100%.

The variables influenced by water retention capacity were analyzed by regression (Figure 1 and Figure 2). An increase in germination was observed with the increase in water availability, reaching maximum values close to the retention capacity of 60%. Under conditions of low water availability (15%), there was a marked reduction in germination.

The formation of abnormal seedlings showed an inversely proportional behavior to water availability (Figure 1B). In general, the increase in water availability promoted increments in emergence speed, first count, growth, and dry mass production of the seedlings. For dead seeds, the increase in the water retention capacity of the substrate led to higher seed mortality (Figure 1C).

Emergence speed index (ESI), first emergence count, shoot length, and shoot dry mass increased with the increase in substrate water retention capacity (WRC). Greater water availability resulted in higher speed and quantity of seedlings emerged, as well as better growth and biomass accumulation. The WRC range between 15% and 30% proved to be more sensitive, as small increments of water promoted significant gains in these variables. Under conditions of lower water availability, an increase in abnormal seedlings and reductions in emergence speed and root growth were observed. Up to 39.59% WRC, the ESI increased more sharply, by 0.175 per unit increase in water, while above this value, the gains were smaller.

A similar behavior was observed for the first emergence count of the seedlings; up to 39.44% WRC, every 1% increase in water availability led to an increase of 2.906% in the percentage of normal seedlings emerged, while for WRC above this most critical level, the increase in the number of normal seedlings is only 0.003 for each 1% increase in water availability (Figure 1E). Root length showed a behavior similar to that of the variables previously presented; up to 37.89% WRC, the increase in root length was 0.38 cm per seedling for each 1% increase in water availability. On the other hand, from 38 to 100% WRC, there was only a 0.035 cm increase in root length per seedling for each 1% increase in water availability (Figure 2B).

Principal component analysis explained 81.01% of the total variance of the data, with 68.48% for the first component and 12.53% for the second component (Table 2).

**Figure 3:**
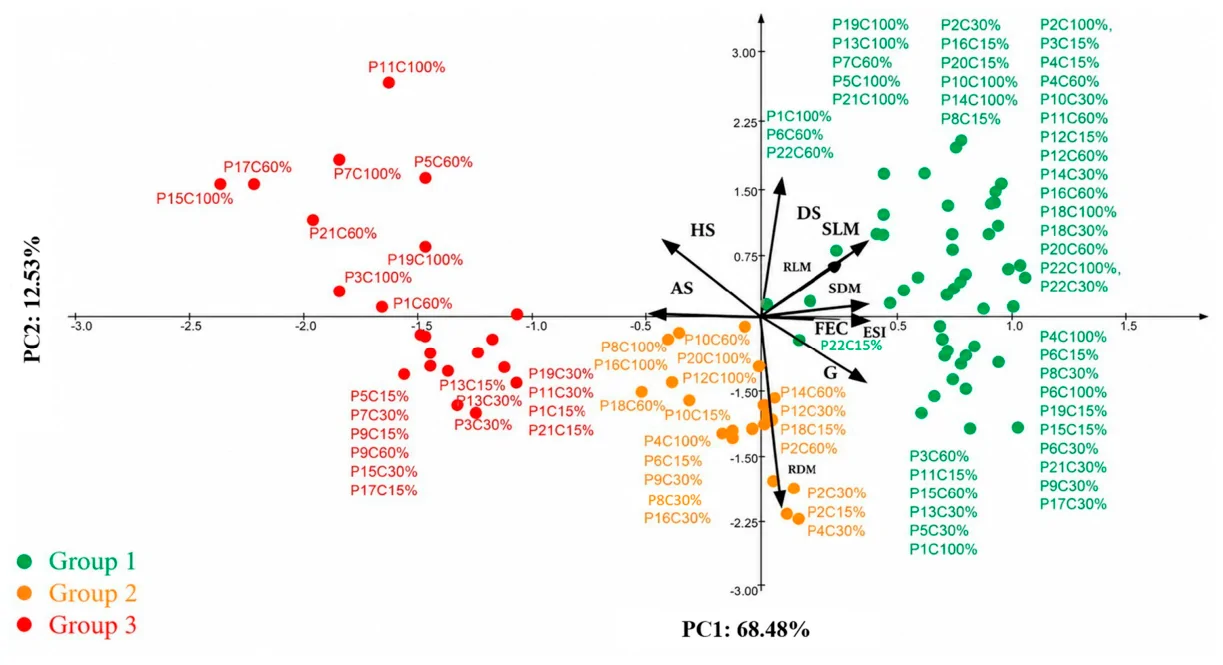
Biplot dispersion plane with eigenvectors obtained from the analysis of two principal components (PC1 and PC2), established based on the variables germination (G), abnormal seedlings (AS), dead seeds (DS), hard seeds (HS), first emergence count (FEC), emergence speed index (ESI), shoot length and shoot dry matter (SL and SDM), and root length and root dry matter (RL and RDM) of 22 maize genotypes subjected to germination under water retention capacities of 15, 30, 60, and 100%.

Cluster analysis in the biplot indicated the formation of three distinct groups, revealing differences in the physiological performance of the genotypes under different conditions of water availability. Group 1 showed the highest germination and vigor of seedlings, i.e., the eigenvectors obtained from these variables are related to the best maize genetic materials at the water retention capacities. Groups 2 and 3 show stronger relationships with the variables’ abnormal seedlings and hard seeds (Figure 3).

### 2.2. Thermal Stress

No germination occurred at a temperature of 5 °C. The *p*-values indicated no effects on abnormal seedlings (*p* = 1.0) and hard seeds (*p* = 1.0), while for dead seeds a significant effect was observed (*p* = 9.10 × 10^−4^ **), with means around 4 to 24%, 75 to 96%, and 0 to 5%, respectively.

The variables hard seeds and abnormal seedlings showed no significant differences at a temperature of 5 °C, ranging from 74 to 96% and 4 to 24%. A significant difference was found in the percentage of dead seeds, forming two groups: the first group containing genotypes 9, 11, 13, 16 to 18, 20, and 21, which had higher percentages of dead seeds, and the second group comprising the others, with lower mortality percentage.

At a temperature of 15 °C (Table 3), a significant difference was observed, with genotypes being classified into three distinct groups for germination. The first group, with the highest germination percentages, was composed of genotypes 6 to 12 and 14 to 19. The second group, with intermediate performance, included genotypes 1 to 5 and 13. The third group, with the lowest germination percentages, was formed by the seeds of genotypes 20, 21, and 22.

At 15 °C, there was a significant difference in abnormal seedlings, forming three groups, and genotypes belonging to Group 1 (6 to 12 and 14 to 19) had lower percentages of abnormal seedlings.

The intermediate group, composed of genotypes 1 to 5 and 13, showed moderate values of abnormal seedlings. In turn, the genotypes of Group 3, which obtained the lowest germination rates, concentrated the highest percentages of abnormal seedlings.

For hard seeds with significant differences, two distinct groups were formed. The first group, with the highest percentage of hard seeds, was represented by material 21, of commercial origin. The other genotypes comprised the second group, with lower percentages of hard seeds.

For dead seeds, a statistical difference was also found in the formation of the two groups. The first, with the highest mortality, was composed of genotypes 10, 13, 16, 18, and 19. On the other hand, genotypes 1 to 9, 11, 12, 14, 15, 17, and 22 showed lower percentages of dead seeds.

Shoot length (Table 3) allowed the formation of four distinct groups with differences from each other. Group 1 was represented by material 1, which had the highest mean of shoot length. Group 2 was composed of genotypes 6, 8 to 11, 14, 15, and 17 to 19. Group 3 included genotypes 2 to 5, 7, 12, 13, and 16, whose seedlings showed intermediate shoot length. Finally, Group 4 was formed by seeds of commercial genotypes 20, 21, and 22, which had the lowest means of seedling shoot length.

Root length was also divided into four distinct groups in the Scott–Knott cluster analysis. The first group was represented by material 19, which had the longest root length. The second group was composed of genotypes 6, 8 to 11, 15, and 17, while the third included genotypes 2 to 5, 7, 12 to 14, 16, and 18, whose seedlings showed intermediate root length. Finally, the fourth group, formed by genotypes 1, 20, 21, and 22, had the lowest mean lengths.

For shoot dry mass, two distinct groups were formed. The first group (represented by the letter a), composed of genotypes 1 to 19, had the highest weights. The second group (referring to the letter b), formed by genotypes 20, 21, and 22, had lower weights.

In the evaluation of root dry mass, these same commercialized genotypes (20, 21, and 22) also showed lower performance, differing statistically from the others. However, for this variable, three distinct groups were formed. The group with the highest accumulation of root dry mass was formed by genotypes 1, 4, 6 to 11, 13 to 15, and 17 to 19. The second group, with intermediate values, included genotypes 2, 3, 5, 12, and 16.

No significant differences were observed in the percentage of germination at 25 °C, ranging from 86 to 99%. The percentages of abnormal seedlings and dead seeds showed no significant difference.

For hard seeds, a significant difference was observed, and two distinct groups were formed: the first, with lower percentages, composed of genotypes 1 to 18 and 20; and the second, formed by genotypes 19, 21, and 22, which had higher percentages of hard seeds (Table 4).

The shoot length formed two groups. The first group, consisting of genotypes 1 to 10 and 12, showed greater development, while the second group, with lower average shoot development, contained genotypes 11 and 13 to 22. A similar behavior occurred for shoot length, with two groups. The first one, formed by the seeds of genotypes 1, 4 to 8, 11, 17 to 20, and 22, had the highest average root length, and the second one, consisting of genotypes 2, 3, 9, 10, 12 to 16, and 21, showed lower values of root length.

Regarding shoot dry mass, two groups were observed. The first, composed of seedlings from genotypes 1 to 18, showed greater dry mass accumulation, while the second group was formed by seedlings of genotypes 19, 20, 21, and 22.

In the evaluation of root dry mass, four groups were formed. The first, with the highest gain in root dry mass, contains material 8, while the second group is composed of 5 and 7, the third group, with intermediate gains in root dry mass, consisted of materials 1, 2, 4, 6, 9, and 10, and the last group comprised the genotypes with the lowest values of root dry mass, 3, 11 to 22.

When the seeds of the genotypes were subjected to 35 °C, no statistical difference was observed for germination (77 and 91%) or dead seeds (0 to 7%) (Table 5).

Under a thermal stress condition of 35 °C, a significant difference was obtained for abnormal seedlings. The first group, composed of genotypes 1 to 3, 6 to 9, and 16 to 18, had the highest percentages of abnormal seedlings. The second group, formed by genotypes 4, 5, 10 to 15, and 19 to 22, showed lower levels of abnormality.

As observed for hard seeds, two distinct groups were identified. The first group, with the highest percentage of hard seeds, consisted of genotypes 21 and 22; the second group was formed by the other genotypes.

For shoot length, two groups were formed: the first group, formed by genotypes 1 to 6 and 9 to 11, and the second group, composed of genotypes 7, 8, and 12 to 22, with lower shoot growth.

For mean root length, four different groups were statistically identified. The first group, formed by genotypes 1 to 5, 10, and 11, had a higher average root length. The fourth group, with the lowest means, consisted of seedlings of genotypes 16, 17, and 19 to 22. Among the intermediate groups, Group 2, with genotypes 6 to 9, 12, and 13, showed higher root lengths than the third group, formed by genotypes 14, 15, and 18.

For shoot dry mass, three groups were formed (a, b, c). The first group, formed by seedlings of genotypes 1, 3 to 5, 9 to 11, 14, 15, and 19, had the highest dry mass accumulation (Group a). The second (Group b), consisting of genotypes 2, 7, 8, 12, 13, and 16 to 18, showed intermediate values, while the third (Group c) exhibited a lower accumulation of shoot dry mass, being composed of genotypes 6, 20, 21, and 22.

Similarly, three groups were also identified for root dry mass. The first, composed of genotypes 1, 3 to 5, and 9 to 15, had the highest mean values. The second group, formed by genotypes 2, 6 to 8, 16 to 19, and 21, showed intermediate results. The third group, consisting of genotypes 20 and 22, had the lowest values.

When maize seeds were subjected to germination at a temperature of 40 °C, there was no germination. The *p*-values indicated no effect on abnormal seedlings (*p* = 1.10^−4^ **) and hard seeds (*p* = 1.10^−4^ **), while for dead seeds a significant effect was observed (*p* = 1.10^−4^ **), with means around 3 to 91%, 0 to 18%, and 7 to 97%, respectively.

For a temperature of 40 °C, the abnormal seedlings variable resulted in the formation of six statistically distinct groups. The first group, represented by material 15, had the highest percentage of abnormal seedlings. The lowest percentage was observed in genotypes 20 and 22. Between these extremes, four intermediate groups were identified: the second group, formed by genotypes 5 and 13; the third group, with genotypes 4, 12 and 14; the fourth group, formed by genotypes 3, 7, 8, 11, 16 and 18; and the fifth group, consisting of genotypes 1, 2, 6, 9, 10, 17, 19 and 21.

Statistical analysis indicated a significant difference between the formation of the two groups for the variable hard seeds. The first group, represented by material 11, had the highest percentage, and the second group, with the lowest percentage, was composed of genotypes 1 to 10 and 12 to 22.

Regarding the variable dead seeds, there was a statistical difference with five groups formed; the first, with the highest percentage of dead seeds, was constituted by genotypes 1, 2, 6, 9, 10, 17, and 19 to 22. The fifth group, with the lowest values of this variable, was formed by genotypes 5 and 15. The intermediate groups comprised the second group (genotypes 3, 7, 8, 16, and 18), the third group (genotypes 4, 11, 12, and 14), and the fourth group (genotype 13).

**Figure 4:**
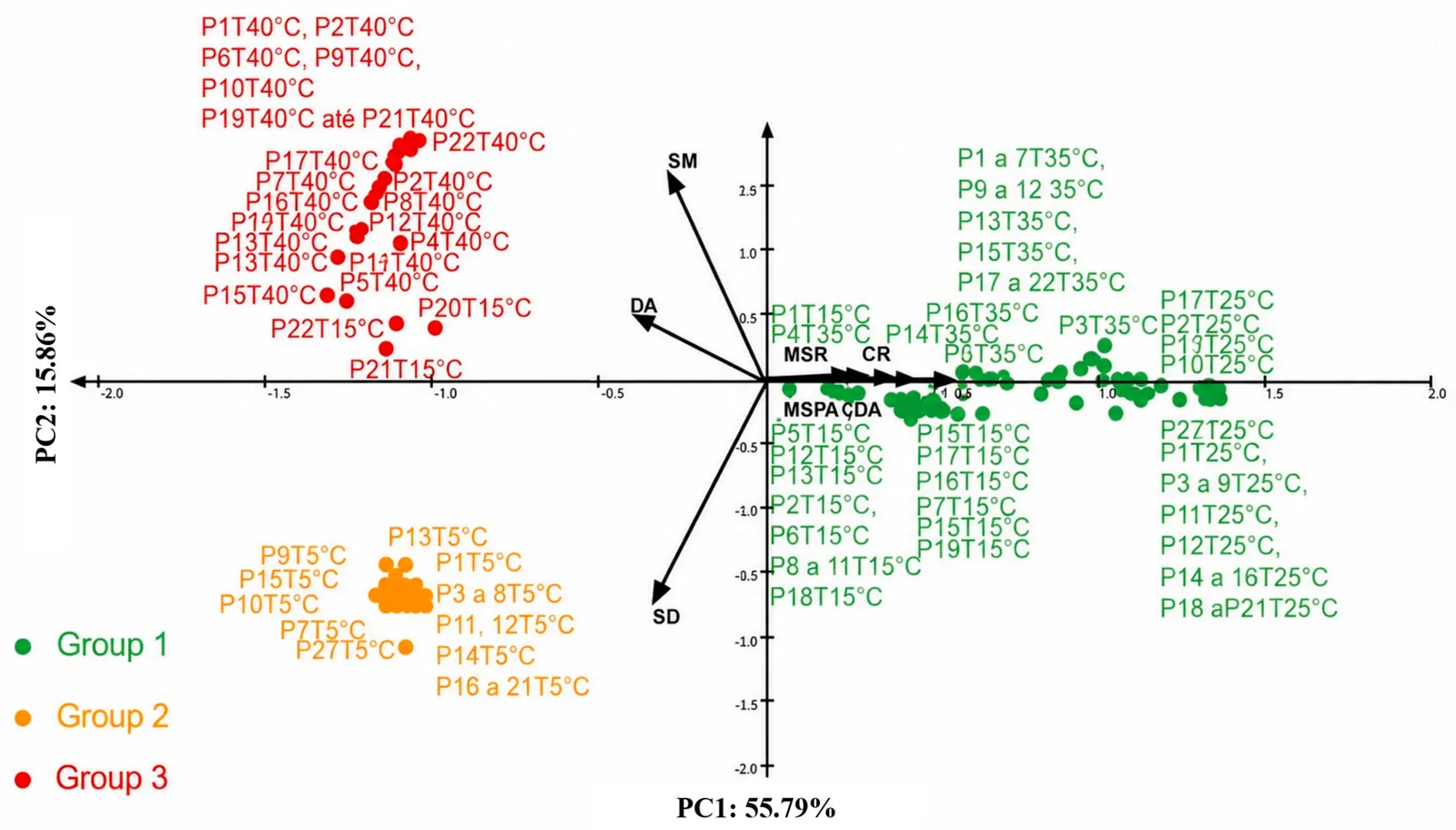
Biplot dispersion plane with eigenvector circle obtained from the analysis of two principal components (PC1 and PC2), established based on the variables germination (G), abnormal seedlings (AS), hard seeds (HS), dead seeds (DS), shoot length and shoot dry matter (SL and SDM), and root length and root dry matter (RL and RDM) of 22 open-pollinated maize genotypes subjected to germination at temperatures of 5, 15, 25, 35, and 40 °C.

In the multivariate analysis, performed by means of the principal component analysis (PCA), as indicated in Table 6 and Figure 4, two components were sufficient to explain the total variability observed among the maize genotypes subjected to different temperatures.

Component 1 explained 55.79% of the total variation, while component 2 explained 15.86%, resulting in a cumulative variance of 71.65%. Similar results were reported by [5,18], who employed this methodology to improve the understanding of the physiological quality of maize seeds as a function of treatments with micro and macronutrients.

In component 1, the variables germination, shoot length, root length, and root dry mass had correlations higher than 0.60, hence being the main responsible for the discrimination of this component. On the other hand, the variables abnormal seedlings, hard seeds, and shoot dry mass had no significant contribution to the explained variation.

For component 2, the variables hard seeds and dead seeds stood out for showing significant correlations (r > 0.60). Between these variables, an inversely proportional relationship was observed. The variables abnormal seedlings and shoot dry mass had no significant discriminatory power in any of the two principal components analyzed.

Principal component analysis (PCA) showed the formation of three distinct groups, represented in Figure 4 (biplot graph) according to the variables evaluated in the different genotypes and at the different germination temperatures.

The first group comprised the seeds of the open-pollinated genotypes 1 to 22 at the temperature of 25 °C, 1 to 22 at 35 °C, and 1 to 19 at 15 °C. This group was mainly associated with component 1, which reflected the best germination rates and the development of seedlings with longer root and shoot lengths, resulting in higher dry mass accumulation for genotypes 1 to 22 at temperatures of 25 and 35 °C, when compared to extreme temperatures of 5 and 40 °C.

With a lower contribution from component 2, these treatments were positioned in the opposite way to the variables related to this component, notably those associated with the occurrence of hard and dead seeds.

The points corresponding to the genotypes maintained at temperatures of 25 and 35 °C were located farther from the point of origin in the biplot graph, indicating that these treatments showed greater germination and better seedling development. Material 3, when subjected to 15 °C, was positioned close to the groups of these two temperatures, although it remained away from the others (Figure 1).

Genotypes 1, 2, and 4 to 19, also evaluated at 15 °C, were grouped in the same set (Group 1), but closer to the point of origin. Although they belong to the same group, some variation is observed between the treatments (Table 3, Figure 1).

Group 2 was composed of open-pollinated genotypes 1 to 22 maintained at 5 °C, located in the opposite quadrant to Group 1. This group showed a higher percentage of hard seeds, mainly explained by component 2. In Group 3, there was also no germination for genotypes 1 to 22 subjected to a temperature of 40 °C. In this same group, genotypes 20 to 22 at 15 °C showed reduced germination, ranging from 6 to 14%, in addition to a high percentage of dead seeds, between 90 and 97% (Figure 1).

## 3. Discussion

### 3.1. Water Stress

The maize genotypes showed variability in germination and vigor, revealing differences in the physiological potential of the seeds. Materials that achieved germination rates above 85% were found to have a lower incidence of abnormal seedlings, hard or dead seeds, and also greater initial growth, indicating better physiological performance. These water stress tolerance characteristics are important to ensure that materials with seed production potential exhibit the same results obtained in laboratory seed quality tests [19,20]. In studies carried out with maize hybrids under water deficit, the characteristics that stood out for resisting the absence of water were related to plant growth [21].

Germination was influenced by water availability, with a marked reduction under conditions of low water retention capacity (15%). This behavior is related to the limitation of imbibition, an essential initial step for the metabolic activation of seeds, including enzymatic processes and mobilization of reserves. Similar results have been observed in wheat [19,22,23], suggesting that low germination can be attributed to the lack of sufficient water to initiate imbibition, causing seed swelling and triggering other metabolic processes with enzymatic and hormonal actions [22,24].

Although germination was not completely inhibited under water restrictions, seedling development was significantly affected by the absence or excess of water. This may be related to the control exerted by water on the continuity of metabolic processes after the beginning of germination, indicating that water availability is more limiting for growth than for germination. The insufficiency of water for the translocation of reserves and nutrients compromises the processes necessary for the formation of normal seedlings [25,26,27]. In a study carried out with the Giza-162 maize hybrid, the authors observed that the low water availability limited plant growth [28]. In evaluations with 28 maize hybrids under a field capacity restriction of 20%, most of the materials showed limitations in development [29]. Ref. [30] studied the effect of water on root cells in maize plants exposed to water stress and reported that there was no change for some treatments, so selecting more tolerant genotypes can be a countermeasure.

With 15% and 30% retention capacity, more time was required for germination processes and seedling establishment, but germination was not completely inhibited. Very similar results were observed for WRC of 60 and 100%. However, the maximum water retention capacity led to greater shoot and root length and dry mass. This result may be related to the fact that, despite the low availability of oxygen, seedlings needed to improve their root development (RL and RDM), possibly to improve respiration efficiency, as also observed in the shoots [31,32].

Some authors report that these increases may be related to a greater nutrient allocation in root tissues [33], rapid osmotic adjustment of root cells, and greater capacity for loosening cell walls [32]. Similar results were observed in maize hybrids, for which a 20% increase in water availability resulted in an increase in yield [34,35].

The accumulated variance observed in this study was 81.01%, higher than that found in tests evaluating the response of maize genotypes to Azospirillum inoculation, 70.67 [36], and lower than that found in [37], which obtained 83.63%, with the use of six principal components.

However, the fact that we were able to achieve an accumulated variance greater than 80% with only two principal components shows that the choice and correlation between the variables have a strong relationship for studies with maize. The technique of principal component analysis is very common to rank maize seed lots with greater vigor and with less loss of information, as corroborated by [5,18], who highlighted the use of this analysis with a correlation of 60% in the ranking of maize seed lots. The higher the accumulated percentage of the variable components, the greater the discriminatory power in the variance of the variables, as reported in a study of genetic diversity of landrace maize in the creation of new materials [38].

Understanding the influence of environmental constraints and morphological, physiological, and chemical responses on crops helps in the development of alternatives to minimize the impact of these stresses [23]. Principal component analysis is a tool that has been contributing greatly to the assays and selection of tolerant genetic populations. Climate change is already projected by the Intergovernmental Panel on Climate Change to have an increase in temperature of up to 2 °C, with a consequent change in rainfall distribution, causing a probable reduction of up to 20% in the production of maize and other crops [8].

The materials studied in the situations of water stress showed germination and development of normal seedlings, so it may be possible to select tolerant materials (3, 4, 8, 12, 16, and 20). Therefore, considering the limitations of germination and vigor imposed by the water deficit within Group 1, genetic materials that showed the best results, some materials stood out precisely for exhibiting similar results to populations that developed under optimal water conditions (60% WRC), namely, 6, 8, 9, 15, 19, and 17.

### 3.2. Heat Stress

Under thermal stress, extreme temperatures directly affected seed performance. At low temperature (5 °C), the imbibition process was delayed, as evidenced by the stiffness of the pericarp and the absence of adequate water absorption. This behavior is related to the reduction in the metabolic activity of the seeds, since low temperatures affect membrane fluidity, promote tissue compaction, and reduce the activity of essential enzymes, such as α-amylase, compromising the degradation of starch and the supply of energy for germination [11].

In contrast, high temperatures (40 °C) resulted in high mortality and lack of development, possibly associated with protein denaturation, reduced enzymatic activity, and non-viability of embryonic tissue. These effects corroborate the results observed in [39], which highlight the unfeasibility of the germination process under high temperatures.

The intermediate temperatures (15 to 35 °C) favored germination and seedling development, indicating a more suitable thermal range for the initial establishment. Although the literature reports limitations on the germination of maize seeds at temperatures below 15 °C, especially in commercial hybrids [40], the genotypes evaluated in this study showed germination means between 91 and 98%. This performance indicates tolerance to suboptimal thermal conditions, although differentiated morphophysiological responses have been observed among the genotypes, such as greater shoot growth associated with reduced root development in some genotypes.

Material 3 showed superior performance, characterized by high germination, greater root and shoot growth, and a greater accumulation of dry mass, maintaining physiological stability even under thermal variation with low and high temperatures, which suggests genetic tolerance to the conditions evaluated.

The principal component analysis (PCA), considering the genotype dataset at different temperatures, revealed an accumulated variance of 71.65%, a value higher than that reported in studies evaluating macro and micronutrients in maize seeds, with approximately 60% [5,18], but lower than that reported [37], who obtained 83.63% using six principal components. These results point to the representativeness of data variability and the strong association between the variables related to the physiological quality of the seeds.

The variable germination, emergence speed index, and first count showed higher factor loadings, which highlights their central role in discriminating the performance of genotypes under different thermal conditions. On the other hand, abnormal seedlings and shoot dry mass had a lower contribution to the total variability, suggesting lower sensitivity of these variables to the temperature variations evaluated [38,41].

Understanding the morphological, physiological, and biochemical responses of seeds to thermal variations is essential to mitigate the impacts of thermal stress on production systems. Temperatures of 15 °C and 35 °C represent typical conditions of the South and Midwest regions of Brazil, respectively, and the maintenance of germination within this wide temperature range indicates that some of the genotypes exhibit physiological stability in the face of temperature variations, although differences in vigor have been observed. The results of this study contribute to the planting positioning of these genotypes [42]. Similar results have been observed [8,43] in studies with wheat and pearl millet under thermal stress.

PCA indicated that the open-pollinated genotypes showed favorable performance under moderate thermal stress (15 °C) when compared to the commercialized genotypes, characterizing them as potential tolerant genotypes, as they proved to be more promising in thermal stress environments. Similar results were reported by [44], whose authors observed delayed development, but not absence of germination, in maize genotypes subjected to temperatures between 15 and 18 °C.

Finally, it was found that the commercialized genotypes showed greater sensitivity to temperatures below 25 °C, possibly due to genetic differences associated with the response to cold, although germination at 15 °C was similar to that observed at 25 °C, considered optimal by [7], indicating wide positioning of these genotypes, diversifying beyond the ideal range of germination. The absence of germination at 40 °C reinforces the importance of selecting genotypes genetically tolerant to a wide range of high temperatures, aiming at the adaptation of the corn crop, since they are probably associated with protein denaturation, membrane disorganization, and irreversible damage to cellular metabolism in adverse climatic scenarios, an important result for the positioning of corn materials.

The variability observed among genotypes, especially among open-pollinated materials, highlights their potential for breeding programs aimed at tolerance to abiotic stresses. The identification of genotypes with stable performance under different conditions reinforces their importance as a source of genetic variability.

## 4. Materials and Methods

### 4.1. Obtaining Seeds and Initial Characterization

A total of 22 maize genotypes from the germplasm bank of the Federal University of Jataí (UFJ), Jatobá Campus, were evaluated. Nineteen non-commercialized maize genotypes were selected, stored in the Genetics Laboratory for 36 months in a cold chamber (10 °C and 45 ± 5% relative humidity), and subsequently transferred to the Seed Laboratory for the experiments. For each material, a working sample of approximately 1 kg was collected. In addition, three varieties already commercialized from the Northeast/Paraíba region of Brazil were implemented in the experiment, following the same storage and conduction criteria as the non-commercialized varieties (Table 7).

Before the application of heat and water stress, in order to assess the quality of the seeds, the genotypes were characterized according to the following determinations and tests:

Moisture content (MC)—determined by the oven method, at 105 ± 3 °C for 24 h, using three subsamples with a proportion following the guidelines of Rules for Seed Analysis (RAS). The procedure was standardized for all capsules, with their bottoms completely covered by the seeds [7].

Thousand-seed weight (TSW)—obtained from eight subsamples of 100 seeds, weighed on an analytical scale with precision of 0.0001 g, according to the criteria established in RAS [7].

Germination (G)—conducted in a Completely Randomized Design (CRD), with six replicates of 25 seeds for each genotype, following a methodology adapted from RAS. The seeds were placed on paper rolls on a layer of sand in order to avoid probable proliferation of microorganisms (creating a physical barrier), and kept in a germination chamber for seven days, under constant photoperiod. On the seventh day, normal seedlings, abnormal seedlings, dead seeds, and hard seeds were counted [7].

Seedling emergence in the field (SEF)—conducted in a Randomized Block Design (RBD), with five blocks, each composed of two rows of 1 m in length for each material, containing 25 seeds per row, sown at approximately 2 cm depth and with a spacing of 5 cm between rows. The blocks were irrigated by sprinklers. Monitoring was carried out daily, and the count was performed as soon as the first seedling emerged after sowing, expressing the results as a percentage.

Emergence speed index (ESI)—calculated based on the daily counts of emerged seedlings, applying the formula proposed [45], IVE = N1/E1 + N2/E2 + N3/E3 +⋯+ Nn/En.

### 4.2. Water Stress

After the initial quality assessment, the genotypes exhibited high physiological quality, with germination ranging from 89% to 98%, ensuring suitable conditions for conducting the experiments [7].

The experiment was conducted in a Randomized Complete Block Design (RCBD) arranged in a 22 × 4 factorial scheme (22 maize genotypes × four levels of water availability: 15, 30, 60, and 100% of the substrate water retention capacity), with four replicates. The water retention capacity of 60% was considered the control condition, according to the Rules for Seed Analysis (RAS) [7]. Four blocks were established to minimize potential environmental variations during the experiment, and the treatments were randomly allocated within each block. Each experimental unit consisted of one acrylic box containing 25 seeds. The assays were carried out under controlled laboratory conditions in B.O.D. germination chambers, with all experimental units maintained under the same temperature and photoperiod conditions throughout the experimental period, thereby minimizing external sources of variation and ensuring the standardization of the control treatment.

Emergence (E) was evaluated with counts of normal seedlings after 12 days of sowing, as described by RAS [7], with adaptation, and the data were expressed as a percentage. Together, the following parameters were evaluated: first emergence count (FEC), with counts of normal seedlings performed on the fourth day after sowing, and emergence speed index (ESI), based on daily counts of normal seedlings [45].

For root length (RL) and shoot length (SL), 10 normal seedlings of each replicate were measured using a graduated ruler after twelve days, and the data were expressed in cm seedling^−1^. For root dry mass (RDM) and shoot dry mass (SDM), seedlings measured for RL and SL were kept under ventilation for 24 h, packed in a kraft paper bag, and subjected to drying in an oven at 65 ± 3 °C for 24 h. Subsequently, the materials were weighed on an analytical scale with 0.0001 g precision, and the results were expressed in g seedling^−1^.

### 4.3. Thermal Stress

The thermal stress assays were conducted in a completely randomized design (CRD), with each temperature evaluated as an independent experiment. The treatments consisted of 22 maize genotypes evaluated at five temperatures (5, 15, 25, 35, and 40 °C), with 25 °C used as the control treatment according to RAS. Each treatment consisted of six replicates, with each replicate corresponding to one paper roll containing 25 seeds. The experimental units were randomly distributed within the germination chambers to minimize possible positional effects [7].

To conduct the tests, the germination papers (Germitest^®^) were previously separated and identified, using three sheets per replicate. Then, they were sterilized in an oven at 105 ± 3 °C for three hours and then moistened with a volume of water equivalent to three times the dry mass of the paper. After moistening, 25 seeds were distributed between the sheets, and rolls were made. The methodology used was adapted according to the one described in RAS, using a 400 g layer of fine sand on the seeds before making the rolls [7].

Germination—normal seedlings, which had all the essential structures for development, and abnormal seedlings, characterized by the absence or malformation of some essential structure, were counted on the seventh day after setting up the test. Dead seeds, when necrosis was observed in the embryo tissues, and hard seeds, the ones that showed a rigid consistency when pressed and did not germinate after the evaluation period, were also recorded. The results were expressed in a percentage (%), according to the criteria established by RAS [7].

Root and shoot length of seedlings—ten normal seedlings from each replicate of the germination test were measured with a graduated ruler, and the results were expressed in centimeters per seedling (cm seedling^−1^) [46].

Root and shoot dry mass of seedlings—seedlings were separated into shoots and roots, cleaned to remove adhered sand, placed in paper bags, and dried in an oven at 65 ± 3 °C for 24 h. Then, they were weighed on an analytic scale (0.0001 g), with the results expressed in g seedling^−1^ [46].

## 5. Statistical Procedure

The assumptions of normality of residuals and homoscedasticity of variances were verified using the Shapiro–Wilk and Bartlett tests, respectively. The data were subjected to analysis of variance (ANOVA) at 1% significance level and, when significant, the means of the qualitative factor were compared by the Scott–Knott means clustering test at 5% significance level.

For thermal stress, univariate statistics were applied, with the variables analyzed separately at each temperature. For water stress, the quantitative factor was subjected to regression analysis, considering linear and non-linear models, with selection based on the Akaike (AIC) and Bayesian (BIC) information criteria [47]. In addition, principal component analysis (PCA) was performed using Past4 version 4.17 software.

## 6. Conclusions

The evaluated maize genotypes showed tolerance to water stress in substrates with water retention capacity ranging from 15% to 100%, maintaining satisfactory germination and seedling development. Materials 3, 4, 8, 12, 16, and 20 showed greater tolerance to water restriction, while material 3 (AS1598) stood out for its superior physiological performance under thermal stress. These results highlight the potential of the evaluated genotypes for use in maize breeding programs aimed at improving tolerance to abiotic stresses.

## Figures and Tables

**Table 1:** Mean values for germination (G), dead seeds (DS), first emergence count (FEC), emergence speed index (ESI), shoot length (SL), and shoot dry matter (SDM) of seeds from 22 maize genotypes.

Materials	G	DS	FEC	ESI	SL	SDM	RDM
	------------%-----------				cm seedling^−1^	g seedling^−1^	
1	85 a	7 a	71 a	4.9 b	17.8 a	0.046 a	0.250 a
2	81 a	1 b	68 a	4.9 b	17.0 a	0.042 a	0.215 b
3	84 a	2 b	75 a	5.2 b	17.0 a	0.042 a	0.230 a
4	85 a	2 b	72 a	5.0 b	17.0 a	0.045 a	0.215 b
5	83 a	3 b	71 a	4.9 b	14.7 b	0.035 b	0.228 a
6	88 a	3 b	74 a	5.3 a	18.3 a	0.043 a	0.194 b
7	86 a	1 b	78 a	5.5 a	17.3 a	0.047 a	0.280 a
8	87 a	3 b	71 a	5.0 b	15.7 b	0.040 a	0.241 a
9	85 a	2 b	76 a	5.8 a	17.6 a	0.044 a	0.230 a
10	82 a	2 b	73 a	5.3 a	17.0 a	0.047 a	0.245 a
11	83 a	6 a	73 a	5.5 a	17.0 a	0.042 a	0.261 a
12	82 a	4 b	72 a	5.1 b	18.1 a	0.045 a	0.223 b
13	76 b	8 a	70 a	5.0 b	17.1 a	0.042 a	0.238 a
14	79 b	6 a	69 a	5.0 b	18.6 a	0.047 a	0.230 a
15	84 a	3 b	69 a	5.0 b	18.5 a	0.045 a	0.252 a
16	71 b	7 a	55 c	4.1 c	13.6 b	0.038 b	0.202 b
17	79 b	5 a	61 b	4.7 c	17.0 a	0.044 a	0.214 b
18	77 b	6 a	62 b	4.6 c	15.6 b	0.044 a	0.255 a
19	70 b	10 a	58 b	4.1 c	15.6 b	0.041 a	0.212 b
20	73 b	10 a	64 b	4.7 c	17.9 a	0.034 b	0.204 b
21	69 b	10 a	51 c	4.1 c	16.3 b	0.032 c	0.216 b
22	83 a	4 b	72 a	5.4 a	17.5 a	0.029 c	0.178 b
M	0.12 **	0.62 **	0.19 **	0.01 **	4.93 **	3.10^−2^ **	6.10^−2^ *
CRA	5.12 **	1.24 **	16.04 **	0.03 *	1613.60 **	0.22 **	1.10^−1^ **
MxCRA	0.02 ^ns^	0.13 ^ns^	0.02 ^ns^	0.11 ^ns^	1.987 ^ns^	0.68 ^ns^	2.10^−2 ns^
CV (%)	12.09	24.03	13.44	20.17	14.71	10.78	8.52

Means followed by the same letter in the column do not differ from each other according to the Scott–Knott mean grouping test at the 5% probability level. * significant at the 5% probability level (*p* < 0.05), ** significant and ^ns^ not significant.

**Table 2:** Correlation of the variables with each principal component and variability of seed quality data from 22 maize genotypes subjected to germination under water retention capacities of 15, 30, 60, and 100%.

Variables	Components 1	Components 2
Germination	0.93	−0.26
Abnormal seedlings	−0.95	−0.00
Hard seeds	−0.81	0.31
Dead seeds	0.22	0.59
First emergence count	0.97	−0.03
Emergence speed index	0.97	0.00
Shoot length	0.91	0.29
Root length	0.95	0.06
Shoot dry matter	0.86	0.26
Root dry matter	0.17	−0.77
Eigenvalue	6.85	1.25
Variance (%)	68.48	12.53
Accumulated variance (%)	81.01	

Component 1 was positively associated with seedling germination, vigor, and growth, and negatively associated with abnormal seedlings and hard seeds. Component 2 had the smallest contribution, particularly for root dry mass.

**Table 3:** Mean values of germination (G), abnormal seedlings (AS), hard seeds (HS), dead seeds (DS), shoot length (SL), root length (RL), shoot dry matter (SDM), and root dry matter (RDM) of seedlings from 22 maize genotypes evaluated individually at a temperature of 15 °C.

Materials	G	AS	HS	DS	SL	RL	SDM	RDM
	------------------%-------------------				cm seedling^−1^		g seedling^−1^	
1	79 b	19 b	0 b	2 b	6.9 a	2.9 d	0.009 a	0.049 a
2	79 b	20 b	1 b	0 b	2.1 c	6.5 c	0.009 a	0.015 b
3	76 b	22 b	1 b	1 b	2.3 c	6.8 c	1.010 a	0.019 b
4	80 b	19 b	1 b	1 b	2.7 c	7.2 c	0.010 a	0.021 a
5	83 b	13 b	1 b	2 b	2.2 c	7.6 c	0.009 a	0.021 b
6	98 a	1 c	1 b	0 b	3.5 b	8.7 b	0.009 a	0.029 a
7	93 a	4 c	3 b	0 b	2.5 c	7.3 c	0.023 a	0.032 a
8	94 a	5 c	1 b	0 b	2.7 b	8.4 b	0.015 a	0.036 a
9	95 a	4 c	1 b	0 b	3.2 b	8.6 b	0.013 a	0.034 a
10	95 a	0 c	0 b	5 a	4.2 b	9.1 b	0.016 a	0.039 a
11	91 a	9 c	0 b	1 b	3.1 b	9.1 b	0.011 a	0.032 a
12	93 a	7 c	0 b	0 b	2.1 c	6.8 c	0.009 a	0.020 b
13	78 b	15 b	3 b	5 a	2.8 c	7.5 c	0.009 a	0.030 a
14	87 a	11 c	2 b	1 b	3.1 b	8.0 c	0.012 a	0.033 a
15	97 a	0 c	2 b	1 b	3.7 b	8.2 b	0.009 a	0.038 a
16	87 a	7 c	2 b	4 a	2.8 c	7.1 c	0.010 a	0.022 b
17	89 a	5 c	5 b	1 b	3.7 b	8.2 b	0.011 a	0.033 a
18	91 a	2 c	1 b	5 a	3.1 b	7.8 c	0.012 a	0.031 a
19	91 a	1 c	5 b	3 a	3.9 b	10.4 a	0.017 a	0.041 a
20	14 c	82 a	3 b	1 b	1.0 d	2.6 d	0.001 b	0.003 c
21	5 c	83 a	11 a	1 b	0.5 d	2.2 d	0.001 b	0.001 c
22	6 c	90 a	4 b	0 b	1.1 d	2.4 d	0.000 b	0.001 c
*p*-value	1 × 10^−4^ **	1 × 10^−4^ **	1 × 10^−4^ **	2 × 10^−4^ **	1 × 10^−3^ **	1 × 10^−3^ **	1 × 10^−2^ **	1 × 10^−3^ **
Standard deviation	10.41	9.89	2.95	2.63	0.78	1.15	0.01	0.01

Means followed by the same letter in the column do not differ from each other according to the Scott–Knott mean grouping test at the 5% probability level. ** significant.

**Table 4:** Mean values of germination (G), abnormal seedlings (AS), hard seeds (HS), dead seeds (DS), shoot length (SL), root length (RL), shoot dry matter (SDM), and root dry matter (RDM) of seedlings from 22 maize genotypes evaluated individually at a temperature of 25 °C.

Materials	G	AS	HS	DS	SL	RL	SDM	RDM
	----------------------%----------------------				cm seedling^−1^		g seedling^−1^	
1	97 a	1 a	0 a	2 a	11.5 a	13.2 a	0.105 a	0.037 c
2	93 a	1 a	0 a	6 a	11.2 a	12.6 b	0.145 a	0.034 c
3	97 a	1 a	0 a	2 a	11.3 a	11.6 b	0.118 a	0.032 d
4	97 a	1 a	0 a	1 a	11.8 a	13.8 a	0.157 a	0.035 c
5	97 a	1 a	0 a	2 a	12.6 a	14.2 a	0.131 a	0.038 b
6	98 a	0 a	0 a	2 a	13.1 a	13.8 a	0.111 a	0.034 c
7	99 a	0 a	0 a	1 a	12.6 a	13.9 a	0.109 a	0.041 b
8	95 a	0 a	0 a	5 a	11.4 a	14.1 a	0.155 a	0.044 a
9	98 a	1 a	0 a	1 a	11.6 a	10.2 b	0.101 a	0.035 c
10	93 a	2 a	0 a	5 a	12.4 a	11.5 b	0.120 a	0.038 c
11	95 a	0 a	0 a	5 a	10.1 b	14.2 a	0.130 a	0.039 d
12	98 a	1 a	0 a	1 a	12.7 a	11.7 b	0.128 a	0.028 d
13	86 a	1 a	0 a	13 a	10.0 b	12.3 b	0.114 a	0.032 d
14	94 a	2 a	0 a	4 a	9.8 b	12.3 b	0.129 a	0.032 d
15	95 a	1 a	0 a	5 a	9.8 b	11.8 b	0.129 a	0.031 d
16	92 a	1 a	0 a	7 a	10.5 b	11.5 b	0.137 a	0.028 d
17	94 a	2 a	0 a	19 a	10.2 b	13.3 a	0.141 a	0.030 d
18	95 a	1 a	0 a	5 a	10.1 b	13.3 a	0.106 a	0.031 d
19	92 a	3 a	1 b	5 a	10.1 b	13.4 a	0.080 b	0.029 d
20	93 a	2 a	0 a	5 a	9.9 b	13.6 a	0.034 b	0.029 d
21	97 a	1 a	1 b	1 a	8.5 b	12.3 b	0.032 b	0.028 d
22	93 a	0 a	3 b	3 a	9.5 b	14.2 a	0.031 b	0.029 d
*p*-value	0.15	0.74	1 × 10^−4^ **	0.22	1 × 10^−3^ **	1 × 10^−3^ **	1 × 10^−3^ **	1 × 10^−3^ **
Standard deviation	9.51	2.20	1.01	9.20	1.74	1.20	0.04	0

Means followed by the same letter in the column do not differ from each other according to the Scott–Knott mean grouping test at the 5% probability level. ** significant.

**Table 5:** Mean values of germination (G), abnormal seedlings (AS), hard seeds (HS), dead seeds (DS), shoot length (SL), root length (RL), shoot dry matter (SDM), and root dry matter (RDM) of seedlings from 22 maize genotypes evaluated individually at a temperature of 35 °C.

Materials	G	AS	HS	DS	SL	RL	SDM	RDM
	---------------------%---------------------				cm plântula^−1^		g plântula^−1^	
1	78 a	17 a	0 b	5 a	6.7 a	10.3 a	0.042 a	0.085 a
2	79 a	17 a	0 b	3 a	7.2 a	10.8 a	0.038 b	0.049 b
3	81 a	18 a	0 b	1 a	7.7 a	11.0 a	0.042 a	0.094 a
4	87 a	9 b	1 b	3 a	7.5 a	9.4 a	0.041 a	0.089 a
5	91 a	7 b	0 b	2 a	7.2 a	9.6 a	0.044 a	0.084 a
6	83 a	17 a	0 b	1 a	7.2 a	8.8 b	0.032 c	0.049 b
7	77 a	21 a	0 b	2 a	6.2 b	8.7 b	0.039 b	0.056 b
8	79 a	19 a	0 b	2 a	6.2 b	8.3 b	0.039 b	0.060 b
9	81 a	16 a	0 b	3 a	7.2 a	8.7 b	0.045 a	0.069 a
10	87 a	11 b	0 b	3 a	7.9 a	9.8 a	0.049 a	0.083 a
11	84 a	13 b	0 b	3 a	7.6 a	9.8 a	0.043 a	0.082 a
12	88 a	12 b	0 b	0 a	6.5 b	8.6 b	0.037 b	0.070 a
13	85 a	13 b	0 b	3 a	6.6 b	8.5 b	0.037 b	0.081 a
14	89 a	7 b	0 b	7 a	5.6 b	7.8 c	0.043 a	0.078 a
15	79 a	9 b	0 b	1 a	6.5 b	7.7 c	0.040 a	0.072 a
16	84 a	18 a	1 b	2 a	5.7 b	6.4 d	0.037 b	0.061 b
17	77 a	11 a	0 b	5 a	5.4 b	6.5 d	0.037 b	0.054 b
18	79 a	20 a	0 b	3 a	6.1 b	7.2 c	0.038 b	0.063 b
19	87 a	7 b	0 b	7 a	6.1 b	6.4 d	0.041 a	0.056 b
20	81 a	13 b	1 b	5 a	5.6 b	6.1 d	0.028 c	0.027 c
21	83 a	7 b	4 a	6 a	5.7 b	5.8 d	0.034 c	0.044 b
22	83 a	9 b	5 a	3 a	6.0 b	6.1 d	0.031 c	0.028 c
*p*-value	0.6 ^ns^	0.1 ^ns^	1 × 10^−4^ **	0.2 ^ns^	1 × 10^−3^ **	1 × 10^−3^ **	1 × 10^−3^ **	1 × 10^−3^ **
Standard deviation	10.42	9.38	1.76	4.20	1.08	1.24	0.01	0.02

Means followed by the same letter in the column do not differ from each other according to the Scott–Knott mean grouping test at the 5% probability level. ** significant and ^ns^ non-significant.

**Table 6:** Correlation of the variables with each principal component and variability of seed quality data from 22 open-pollinated maize genotypes subjected to germination at temperatures of 5, 15, 25, 35, and 40 °C.

**Variables**	**Components 1**	**Components 2**
Germination (G)	0.97	0.00
Abnormal seedlings (AS)	−0.58	0.22
Hard seeds (HS)	−0.56	−0.79
Dead seeds (DS)	−0.48	0.78
Shoot length (SL)	0.92	0.01
Root length (RL)	0.97	0.00
Shoot dry matter (SDM)	0.42	0.03
Root dry matter (RDM)	0.82	0.02
Eigenvalue	4.46	1.27
Variance (%)	55.79	15.86
Accumulated variance (%)	71.65	

**Table 7:** Description of the open-pollinated maize genotypes and the commercialized varieties.

Identification	Origin	Genetic Coding and Description
1	Goiás	RB9210—Recurrent selection in half-sib families evaluated at the experimental farm of Federal University of Jataí and, after selection, recombined.
2	Goiás	RB9210—Recurrent selection in half-sib families evaluated under farmer field conditions and, after selection, recombined.
3	Goiás	AS1598—Recurrent selection in half-sib families evaluated at the experimental farm of Federal University of Jataí and, after selection, recombined.
4	Goiás	AS1598—Recurrent selection in half-sib families evaluated under farmer field conditions and, after selection, recombined
5	Goiás	AS1598—F2 seeds originating from a single-cross hybrid production field, recombined.
6	Goiás	RB9210—F2 seeds originating from a single-cross hybrid production field, recombined.
7	Goiás	RV Cercospora Composite—Selection of half-sib families based on the Williams base selection index and, after selection, recombination.
8	Goiás	RV Cercospora Composite—Selection of half-sib families based on the classical Smith and Hazel index and, after selection, recombination.
9	Goiás	RV Cercospora Composite—Selection of half-sib families based on the Rank Sum index of Mulamba and Mock, and after selection, recombination.
10	Goiás	RV Cercospora Composite—Selection of half-sib families based on mixed models—BLUP and, after selection, recombination.
11	Goiás	RV Cercospora Composite—Selection of half-sib families based on inbreeding depression (FS1) and, after selection, recombination of the corresponding half-sib families.
12	Goiás	Cross CRE02 × RB9210 with recombination of the generated families containing 50% introgression of exotic germplasm (CRE02) and recombination of the generated families.
13	Goiás	Cross HEAT SELECTED COMPOSITE × P3646 with 50% introgression of exotic germplasm (heat composite) and recombination of the generated families.
14	Goiás	Cross CRE02 × P4285 with 50% introgression of exotic germplasm (CRE02) and recombination of the generated families.
15	Goiás	Cross NAP–HT (Exserohilum turcicum) × CD384 with 50% introgression of exotic germplasm (NAP-HT) and recombination of the generated families.
16	Goiás	Originated from the cross between the commercial hybrid AG1051 and the synthetic TG02R2, with selection for production-related traits, emphasizing pamonha production.
17	Goiás	Originated from the cross between the commercial hybrid AG1051 and the synthetic TG02R2, with selection for physical traits focusing on ear diameter, number of rows, row alignment, length, husk coverage, shape, usable length, and lower caterpillar damage to ears.
18	Goiás	Originated from the cross between the commercial hybrid AG1051 and the synthetic TG02R2, with selection for production-related traits focused on fresh consumption, including usable weight and ear weight without husk, length, diameter, number of rows, ear alignment, kernel color, and lower caterpillar damage.
19	Goiás	Originated from the cross between the commercial hybrid AG1051 and the synthetic TG02R2, with selection focused on ear diameter and length, biomass production, number of ear rows, and caterpillar damage.
20 to 22	Paraíba	Commercial materials: AL Bandeirante, AL Piratininga, and AL Paraguaçu.

## Data Availability

The original contributions presented in this study are included in the article. Further inquiries can be directed to the corresponding author.
